# Draft genome sequence of halotolerant plant growth-promoting *Bacillus paralicheniformis* MHN12

**DOI:** 10.1128/mra.01138-23

**Published:** 2024-03-04

**Authors:** Priyanka Dahiya, Pradeep Kumar, Simran Rani, Amita Suneja Dang, Pooja Suneja

**Affiliations:** 1 Plant-Microbe Interaction Laboratory, Department of Microbiology, Maharshi Dayanand University, Rohtak, Haryana, India; 2 Centre for Medical Biotechnology, Maharshi Dayanand University, Rohtak, Haryana, India; The University of Arizona, Tucson, Arizona, USA

**Keywords:** whole-genome sequencing, *Bacillus paralicheniformis*, halotolerant, plant growth promotion, secondary metabolites

## Abstract

*Bacillus paralicheniformis* MHN12 possesses a 4,245,453-base pair genome with 45.9% G + C content, including 1 CRISPR, 80 tRNA, 8 rRNA genes, and 4,418 predicted coding sequences . MHN12 exhibits high salinity tolerance and plant growth-promoting abilities, making it a promising bioinoculant for enhancing plant growth in saline soils.

## ANNOUNCEMENT

Legumes are crucial to global food security due to their high protein and nutrient content. However, climate change, abiotic stresses like salinity, and diseases like Fusarium wilt caused by *Fusarium oxysporum* impact their yield consistency. Current management methods involve synthetic fungicides, leading to environmental issues (1, 2). Stress-resilient plant growth-promoting endophytic bacteria offer an eco-friendly solution for sustainable plant growth and protection (3). The use of beneficial native microbes for promoting plant growth and protection is favored, as foreign strains often struggle to colonize and benefit host plants (4).

**Fig 1:**
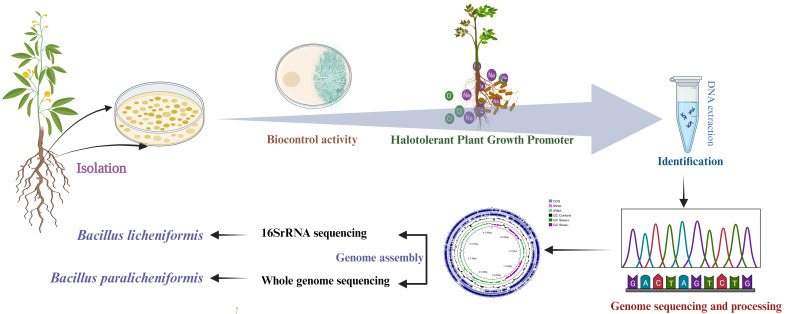
Genome sequencing of halotolerant plant growth-promoting *Bacillus paralicheniformis* MHN12.

The isolation of *Bacillus paralicheniformis* MHN12 was conducted from *Vigna radiata* nodules collected from Hisar, Haryana, India (29.25°N, 76.05°E) with the aim to identify a stress-resilient plant growth-promoting endophyte. The nodules were surface sterilized (5), crushed, and streaked onto Tryptic Soya Agar plates. The streaked plates were kept for 3 days at 28°C ± 2°C to allow bacterial growth. Non-macerated sterilized nodules served as a control to validate the effectiveness of the sterilization process. Genomic DNA from selected pure colonies was extracted by a modified cetyltrimethylammonium bromide protocol (6). The extracted DNA was then amplified by 16S rRNA universal primers (1541R, 8F) (7) followed by sequencing. The obtained sequences were compared using the BLAST server and submitted to GenBank (MH298522). Based on 16S rRNA sequence similarity, MHN12 showed the highest affiliation to *Bacillus licheniformis*. The isolate exhibited plant growth-promoting traits, including phosphate solubilization, 1-aminocyclopropane-1-carboxylic acid deaminase activity, hydrogen cyanide, siderophore, and indole-3-acetic acid production. It demonstrated high salinity tolerance (15%) and antifungal activity against *Fusarium oxysporum and Aspergillus niger* (8, 9). Therefore, it prompted us to look deeper into its whole genome (Fig. 1) .

The paired-end sequencing library was constructed using the Illumina TruSeq Nano DNA Library Prep Kit and sequenced on an Illumina NextSeq500 platform with 2 × 150 bp chemistry. The whole-genome sequencing generated 1,437,736,607 bases, providing around 100× genome coverage. For genome analysis and annotation Galaxy server tools, Prokka v1.14.5 (10), Shovill v1.0.4 (11), and Fastp v0.201 (12) with default parameters were utilized. The filtering and trimming using Fastp v0.201 yielded 5,043,632 paired ends, assembled into 32 contigs by Shovill v1.0.4, generating an assembly of 4,245,453 bp with N50 of 503,829 bp. Genome annotation by Prokka v1.14.5 revealed genome harbors 1 CRISPR gene, 80 tRNA genes, 8 rRNA genes, and 45.9% GC content. Additional rapid annotation subsystem technology (13) annotation identified 4,418 predicted coding sequences (CDS) across 480 subsystems. Abundant subsystems included carbohydrates (598 CDS), amino acids and derivatives (484 CDS), and cofactors, vitamins, prosthetic groups, and pigments (246 CDS). AntiSMASH v6.0 (14) analysis revealed secondary metabolite regions (fengycin, bacitracin) in MHN12’s genome. Despite initial identification as *licheniformis*, subsequent sequencing and annotation supported its reclassification as *paralicheniformis*, confirmed by the presence of specific secondary metabolite operons as reported in previous studies (15).

The draft genome of *Bacillus paralicheniformis* MHN12 provides crucial insights, serving as a foundation to explore the mechanisms for diverse plant growth promoting and stress resilience. Also, this knowledge contributes to the development of eco-friendly strategies for enhanced legume cultivation and improved yields.

## Data Availability

*Bacillus paralicheniformis* MHN12 whole-genome sequence has been deposited at GenBank under accession no. GCA_024464535.1 for the chromosome and annotated by the prokaryotic genome annotation pipeline (16). The BioProject accession no. is PRJNA848808 (Sequence Read Archive accession no. SRR19708069).
